# Spatial Surface Charge Engineering for Electrochemical Electrodes

**DOI:** 10.1038/s41598-019-51048-5

**Published:** 2019-10-10

**Authors:** Lingyun Xie, Peng Wang, Yinping Qian, Lujia Rao, Hongjie Yin, Xingyu Wang, Hedong Chen, Guofu Zhou, Richard Nötzel

**Affiliations:** 1 Guangdong Provincial Key Laboratory of Optical Information Materials and Technology, South China Academy of Advanced Optoelectronics, South China Normal University, Guangzhou, 510006 P.R. China; 2 National Center for International Research on Green Optoelectronics, South China Normal University, Guangzhou, 510006 P.R. China; 3 Academy of Shenzhen Guohua Optoelectronics, Shenzhen, 518110 P.R. China

**Keywords:** Materials science, Nanoscience and technology

## Abstract

We introduce a novel concept for the design of functional surfaces of materials: Spatial surface charge engineering. We exploit the concept for an all-solid-state, epitaxial InN/InGaN-on-Si reference electrode to replace the inconvenient liquid-filled reference electrodes, such as Ag/AgCl. Reference electrodes are universal components of electrochemical sensors, ubiquitous in electrochemistry to set a constant potential. For subtle interrelation of structure design, surface morphology and the unique surface charge properties of InGaN, the reference electrode has less than 10 mV/decade sensitivity over a wide concentration range, evaluated for KCl aqueous solutions and less than 2 mV/hour long-time drift over 12 hours. Key is a nanoscale charge balanced surface for the right InGaN composition, InN amount and InGaN surface morphology, depending on growth conditions and layer thickness, which is underpinned by the surface potential measured by Kelvin probe force microscopy. When paired with the InN/InGaN quantum dot sensing electrode with super-Nernstian sensitivity, where only structure design and surface morphology are changed, this completes an all-InGaN-based electrochemical sensor with unprecedented performance.

## Introduction

Electrochemical electrodes are prime examples of materials with functional surfaces. One of their key applications is as electrochemical sensors. Electrochemical sensors are used in medical diagnostics^1,2^, environmental monitoring^3,4^, food safety analysis^5^, industrial process control^6^ and bio-defense^7^. They are conceptually robust, fast, simple to use on-site in real-time and cheap. Every electrochemical sensor, amperometric^8^ or potentiometric^9,10^, requires a reference electrode next to the sensing electrode^11–14^ to set a constant potential. While there are many robust, all-solid-state solutions for the sensing electrode, the reference electrode almost always is the liquid-filled Ag/AgCl electrode. It consists of a chlorinated Ag wire immersed in a saturated KCl solution, which is in contact with the analyte solution through a porous material. Such electrodes are bulky and difficult to miniaturize. They are not compatible with planar device fabrication technology, have a limited lifetime and need cumbersome maintenance. Conventional liquid-filled reference electrodes need to be replaced for realizing compact and robust electrochemical sensors and sensor arrays. Simply using inert noble metal wires of Ag or Pt^15,16^ as quasi-reference electrodes does not result in a stable response. More promising is the reduction of the sensitivity of all-solid-state sensing electrodes through passivation of active sites using, e.g., silanes^17^, PVC^18^ or polypyrrole^19^. However, reduction of the sensitivity to a target below 10 mV/decade of analyte concentration with acceptable drift below 5 mV/hour is difficult.

In search for the best materials, InGaN has recently been uncovered for electrochemical sensor electrodes due to its high chemical stability, non-toxicity and bio-compatibility^20–22^. In addition, InN/InGaN quantum dot (QD) ion- and bio-sensing electrodes have been discovered with unprecedented, super-Nernstian potentiometric response^23–26^. The superior performance has been assigned to the presence of a high density of intrinsic, positively charged donor states on the InN surface^27,28^ together with the zero-dimensional electronic properties of the QDs^26^. These sensing electrodes have been operated together with conventional Ag/AgCl reference electrodes, limiting the realization of compact sensor devices.

Here we demonstrate a stable, all-solid-state, epitaxial InN/InGaN-on-Si reference electrode upon a sophisticated interplay of structure design, surface morphology and surface charge. Such an all-solid-state reference electrode allows compact and robust, easy to maintain all-solid-state miniaturized sensors and sensor arrays, removing all the difficulties associated with liquid-filled reference electrodes. The basic idea is to create a surface with balanced surface charge distribution at the nanoscale, i.e., balanced nanoscale regions with positive and negative surface charge, such that a stable, analyte concentration insensitive response originates. This exploits the unique surface properties of InGaN: (i) transition from negatively to positively charged surface states at about 45% In content for the c-plane, reaching a density of 2 × 10^13^ cm^−2^ for InN^29,30^ and (ii) negatively charged surface states for the m-plane over the entire In composition range^28^. The parameters for electrode construction at hand are the In content of the InGaN layer, the amount of deposited InN and the InGaN surface morphology, which is determined by the growth conditions (growth temperature and V-III flux ratio) and layer thickness. For the right parameters the reference electrode exhibits less than 10 mV/decade potential response over a wide concentration range from 0.001 to 1 M KCl aqueous solutions and less than 2 mV/hour long-time potential drift over 12 hours in 0.1 M KCl aqueous solution. The delicate interplay of structure, morphology and surface charge for the electrochemical response is underpinned by measurements of the relative surface potential by Kelvin probe force microscopy (KPFM). This introduces the concept of spatial surface charge engineering for achieving the desired surface functionality. By solely modifying structure and morphology and, hence, surface charge distribution, the highly sensitive InN/InGaN QD sensing electrode is obtained, complementing the concept.

In a broader view, next to epitaxial III-V semiconductor heterostructures, surface and interface engineering also plays an important role in improving the properties of the heterostructures of metal oxides, two-dimensional materials and nanoparticles. As for epitaxial III-V semiconductors and specifically nitride semiconductors^26,31,32,^ applications include catalysis^33,34^, photovoltaics^35^, memories^36,37^, and photodetectors^38–43^. Various combinations of different materials have been realized and design parameters include doping, lattice matching, energy band matching and metal particle decoration. Certainly these approaches provide complementary solutions.

For reference electrode fabrication, facing a vast amount and combinations of available parameters, we pre-select an In content of the InGaN layer of 45%, where a charge neutral c-plane surface with concentration insensitive but unstable response is expected. InGaN layers with lower or higher In content exhibit strong response due to a high density of either negatively or positively charged surface states. In particular this has been reported for pure GaN and InN layers^22^. We discuss only samples fabricated at a growth temperature close to the InGaN decomposition temperature and with close-to-stoichiometric V-III flux ratio, as only these conditions lead to surface morphologies with concentration independent response. Planar InN/InGaN layers grown on GaN/sapphire substrates always show strong response^23–25^. Hence, the key parameters explicitly highlighted are the InN coverage and the thickness of the InGaN layer grown on Si. Doping might be another design parameter, however, because of the high n-type conductivity of the InGaN layers due to a high density of defects, acting as donors, doping is expected to have minor influence. On the other hand, these defects do not appear detrimental for the functionality of the electrodes, which is purely surface related. Best parameter identification is done for cycling in 0.1 and 1 M KCl aqueous solutions. The best candidate is then evaluated over a wide concentration range and long time.

## Results

### OCP as function of InN deposition amount

**Figure 1:**
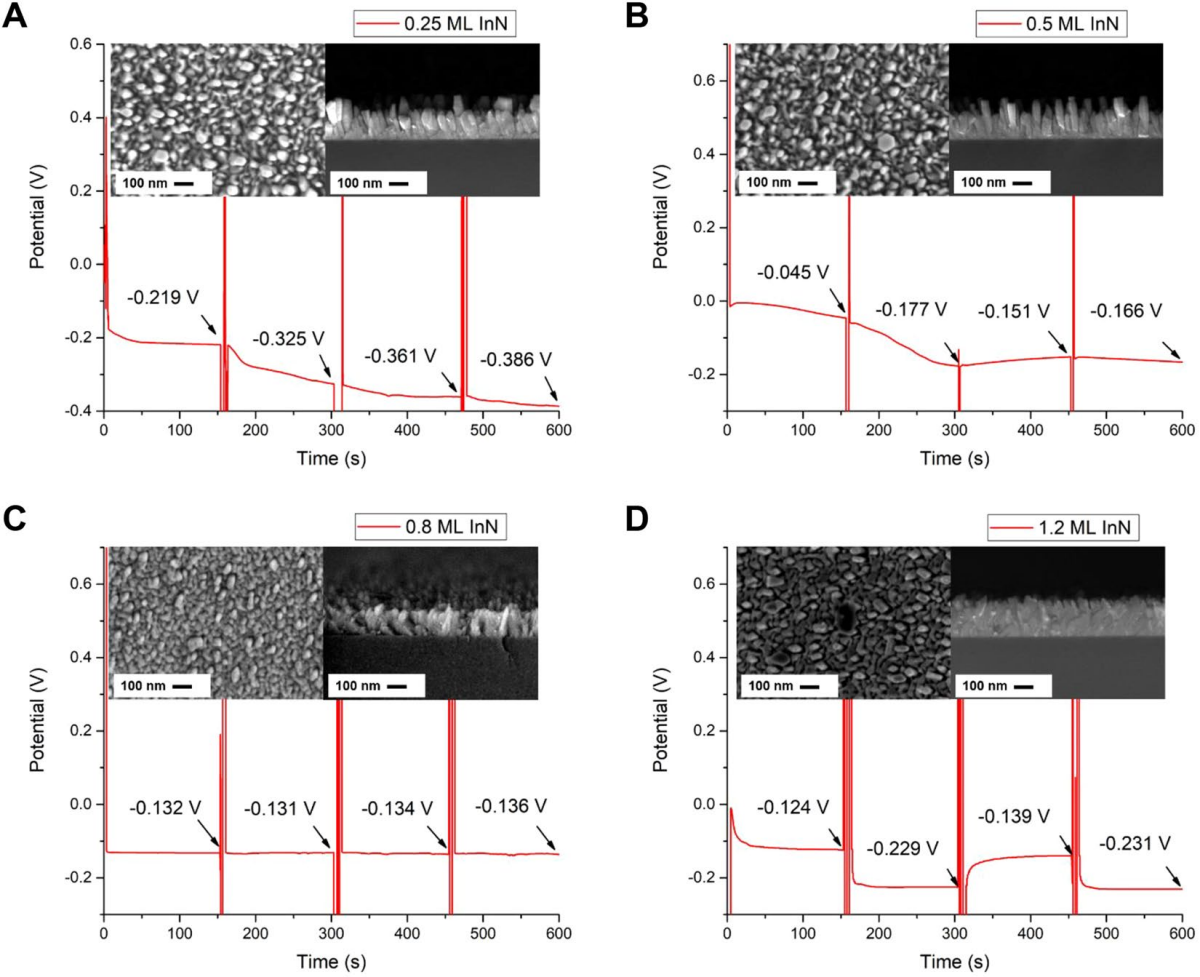
Open circuit potential as function of InN coverage. OCP as a function of time for the InN/InGaN electrode and Ag/AgCl reference electrode immersed alternately for 150 seconds in 0.1 and 1 M KCl aqueous solutions. The insets show the top-view (left-hand side) and cross-sectional (right-hand side) SEM images. The InN deposition amounts are (**A**) 0.25, (**B**) 0.5, (**C**) 0.8 and (**D**) 1.2 ML. The thickness of the In_0.45_Ga_0.55_N layer is 95 nm for all samples.

Figure 1 shows the open circuit potential (OCP) as a function of time for the InN/InGaN samples with 95 nm thick InGaN layers and 0.25 (Fig. 1A), 0.5 (Fig. 1B), 0.8 (Fig. 1C) and 1.2 (Fig. 1D) monolayer (ML) InN deposition amount. The InN/InGaN samples are alternately immersed for 150 seconds in 0.1 and 1 M KCl aqueous solutions. The insets show the top-view scanning electron microscopy (SEM) images (left-hand side) and cross-sectional SEM views (right-hand side) of the respective InN/InGaN structures. The cross-sectional images reveal dense, columnar growth with the columns touching and eventually coalescing. On the sample surfaces this translates into an undulated surface morphology, seen in top-view SEM. Within develop individual, isolated columns, which are distributed randomly. This is due to the complex nucleation behavior of In-rich InGaN directly on Si, which is not discussed further here. The InN layers cannot be distinguished on these non-planar surfaces. For the 0.25 and 0.5 ML InN layers, the OCP shows a weak potential response directly after immersing the InN/InGaN samples into the KCl solutions with different concentrations. Afterwards, however, there is a large drift. This drift is strongly reduced for the 0.8 ML InN layer, being a good candidate for a reference electrode. To note, the 0.25, 0.5 and 0.8 ML InN coverages are below or just at the critical thickness for QD formation on planar InGaN layers grown on GaN/sapphire substrates, shown in Supplementary Fig. S1 together with the OCP response. In marked contrast, for the 1.2 ML InN coverage, where QDs are formed on planar InGaN layers on GaN/sapphire, shown in Supplementary Fig. S2 together with the OCP response, the OCP shows a large and stable potential response of 90–100 mV/decade of KCl concentration, revealing the InN/InGaN QD sensing electrode with super-Nernstian response.

### OCP as function of InGaN layer thickness

**Figure 2:**
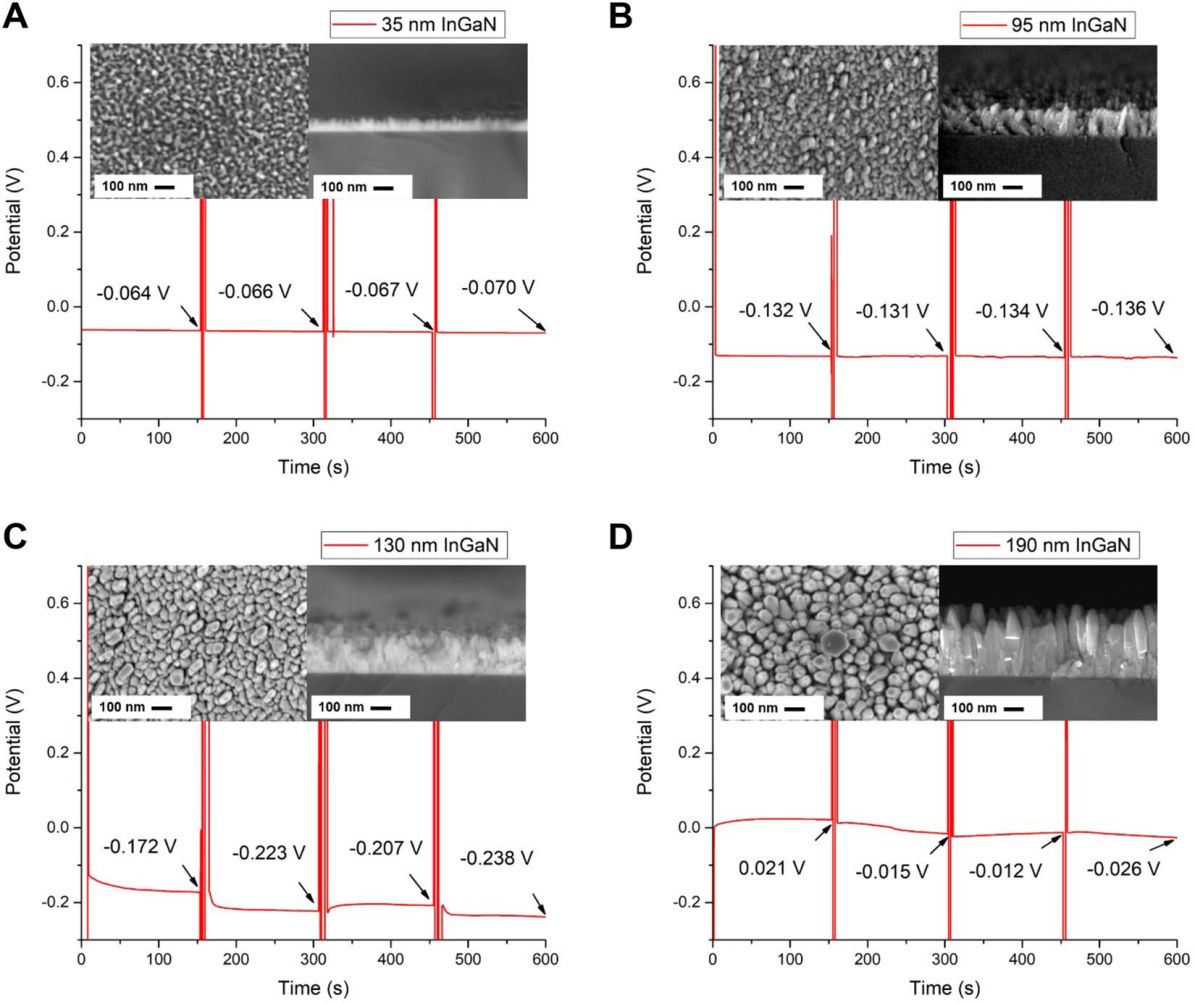
Open circuit potential as function of InGaN layer thickness. OCP as a function of time for the InN/InGaN electrode and Ag/AgCl reference electrode immersed alternately for 150 seconds in 0.1 and 1 M KCl aqueous solutions. The insets show the top-view- (left-hand side) and cross-sectional (right-hand side) SEM images. The In_0.45_Ga_0.55_N layer thicknesses are (**A**) 35, (**B**) 95, (**C**) 130 and (**D**) 190 nm. The InN deposition amount is 0.8 ML for all samples.

Figure 2 shows the OCP measurements as a function of time for the InN/InGaN samples where the InGaN layer thickness is varied for the fixed, best 0.8 ML InN coverage. The InGaN layer thickness is 35 (Fig. 2A), again 95 (Fig. 2B), 130 (Fig. 2C) and 190 nm (Fig. 2D). The InN/InGaN samples are alternately immersed for 150 seconds in 0.1 and 1 M KCl aqueous solutions. The insets show the corresponding top-view and cross-sectional SEM images. The columnar structure seen in cross-sectional SEM is observed for all samples with the undulated surface morphology visible in top-view SEM. The individual, isolated columns, seen in top-view SEM, grow in height and diameter and dominate with increasing layer thickness. They are almost invisible for the 35 nm thick InGaN layer. In the OCP measurements, the 0.8-ML-InN/35-nm-InGaN sample and the 0.8-ML-InN/95-nm-InGaN sample show comparable low response towards the KCl concentration with high stability. The thicker 130- and 190-nm-InGaN samples develop a clear response towards the KCl concentration and large drift. Because of the technological- and cost relevance of thin layers, we choose the 0.8-ML-InN/35-nm-InGaN sample to evaluate the low sensitivity for a wide KCl concentration range and the high long-time stability required for a reference electrode.

### OCP over a wide concentration range and long time

**Figure 3:**
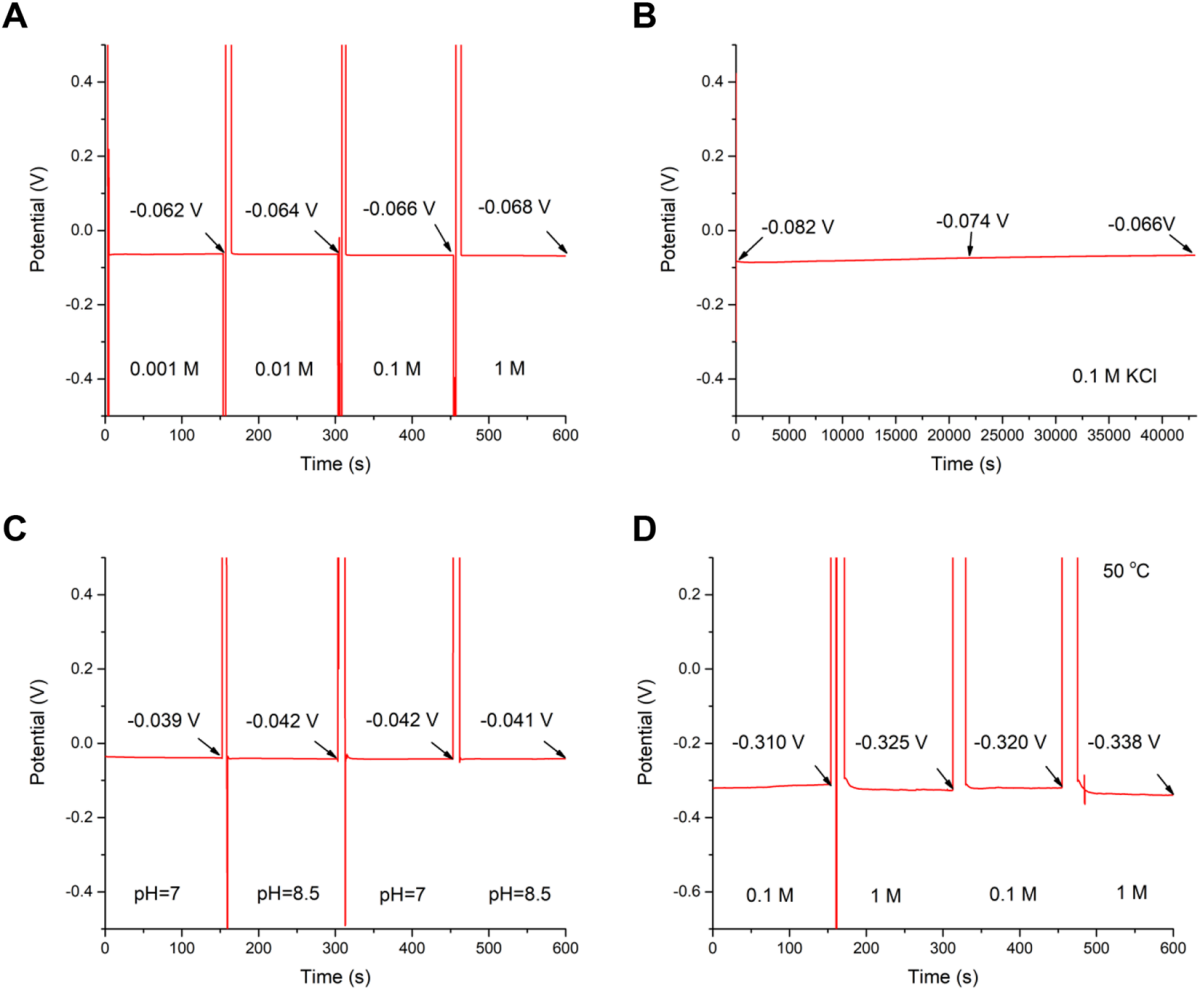
Open circuit potential for the InN/InGaN reference electrode. (**A**) OCP as a function of time for the 0.8-ML-InN/35-nm-In_0.45_Ga_0.55_N electrode and Ag/AgCl reference electrode successively immersed for 150 seconds in 0.001, 0.01, 0.1 and 1 M KCl aqueous solutions. (**B**) Long-time OCP measurement for the same sample over 12 hours in 0.1 M KCl aqueous solution. (**C**,**D**) OCP as a function of time for the 0.8-ML-InN/35-nm-In_0.45_Ga_0.55_N electrode and Ag/AgCl reference electrode immersed alternately for 150 seconds (**C**) in water and pH 8.5 KOH aqueous solutions and (**D**) in 0.1 and 1 M KCl aqueous solutions at 50 °C.

Figure 3A shows the OCP measurements as a function of time for the InN/InGaN sample with 0.8 ML InN coverage and 35 nm InGaN layer thickness successively immersed for 150 seconds in 0.001, 0.01, 0.1 and 1 M KCl aqueous solutions. Over the whole concentration range the potential response with drift is less than 10 mV, satisfying the demands on a reference electrode. Also the long-time OCP response, shown in Fig. 3B, qualifies the 0.8-ML-InN/35-nm-InGaN sample as a reference electrode with a low drift of less than 2 mV/hour over 12 hours in 0.1 M KCl aqueous solution.

After having identified the best reference electrode tested in KCl aqueous solution we also cross-checked in KOH alkaline solution and at elevated temperature. In acidic solutions, the InN/InGaN electrodes inherently show no response to the positively charged hydrogen ions^24^. In Fig. 3C,D the corresponding measurements are shown for cycling alternately for 150 seconds between water and pH 8.5 KOH aqueous solution (Fig. 3C) and between 0.1 and 1 M KCl aqueous solutions at 50 °C (Fig. 3D). The reference electrode behaves equally well in KOH aqueous solutions. At 50 °C it is slightly less stable, but still acceptable. Corrosion is neither observed in alkaline nor in acidic solutions^24^.

## Discussion

**Figure 4:**
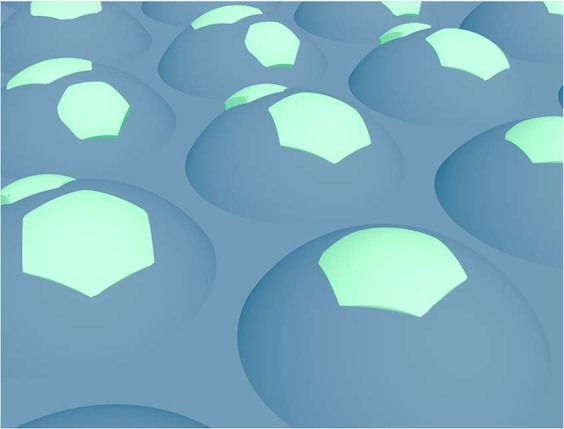
Spatial surface charge engineering. Graphics of the undulated InGaN surface with sub-ML InN coverage. The sidewalls are negatively charged and the InN ML-islands on top are positively charged.

From the experimental results, two main conclusions follow: (i) there is an optimum InN coverage and (ii) there is an optimum surface morphology. As outlined, the basic idea is to create a surface with balanced surface charge distribution at the nanoscale. This clearly calls for a particular surface morphology covered with a particular amount of InN. We started to imagine a c-plane In_0.45_Ga_0.55_N layer having a planar surface, which is charge neutral. However, a charge neutral surface is not expected to produce a stable response. But any evolution of surface morphology will expose sidewalls with negative surface charge while sub-ML deposition of InN will add positive surface charge on top. Both together, rightly balanced, will give rise to the nanoscale regions with positive and negative surface charge for concentration insensitive and stable electrochemical response. The band structure of the InGaN/InN heterostructure has been drawn in ref.^44^ in the context of photoelectrochemical water splitting, including the pulling down of the surface band bending upon deposition of InN. This describes the attraction of electrons inside the InGaN layer to the surface due to the positive surface charge of InN. Figure 4 shows a graphics of the undulated InGaN surface originating from the columnar growth, partially covered with InN in form of ML-islands. Away from the optimum distribution will be samples with low response but large drift or high response and small or large drift, as observed experimentally. This is correlated with the KPFM measurements providing direct access to the changes of the surface charge.

**Figure 5:**
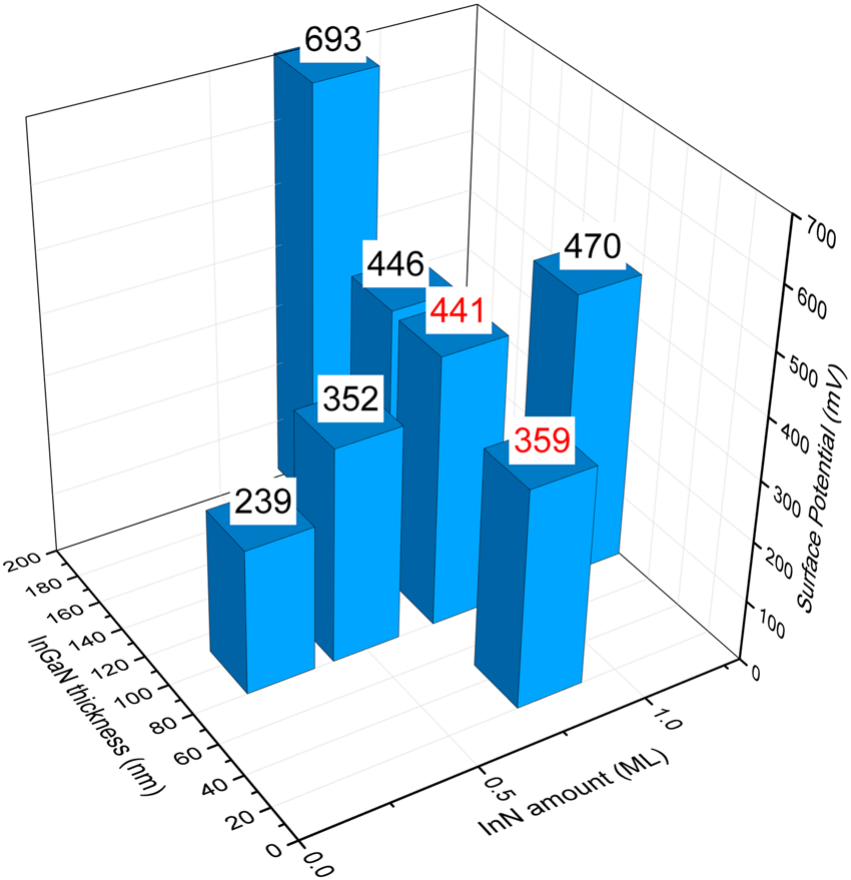
Correlation with surface potential. Diagram of the relative surface potentials measured by KPFM of the electrodes as a function of the InN deposition amount and InGaN layer thickness. Labeled in red are the best reference electrodes.

Figure 5 shows a diagram of the relative surface potentials measured by KPFM of the electrodes as a function of the InN deposition amount and InGaN layer thickness. The KPFM resolution of 100 nm allows only average values. The nanoscale surface features are not captured in the relative surface potential. The KPFM measurements are performed under illumination to obtain a stable and reproducible signal. In the dark, random fast and slow charging and discharging processes at the surface turn the measurements unreliable^45,46^. Such processes are screened and stabilized under illumination. As a function of InN amount the surface potential continuously increases. This evidences the subsequent addition of positive surface charge with increasing amount of deposited InN. Also with increase of the InGaN layer thickness the surface potential and, hence, positive surface charge increases. This is attributed to the change of the surface morphology. With increasing InGaN layer thickness, the isolated columns exposing c-plane top facets develop. These c-plane top facets, covered with InN, add positive surface charge and reduce the negative surface charge associated with the undulated surface morphology, diminishing around the developing columns. Altogether, the red labeled samples with intermediate surface potentials are most concentration insensitive and stable in the OCP measurements. Also these samples are neighbors in a row as a function of the surface potential, no matter if the change is due to the InN amount or the InGaN layer thickness/surface morphology. For larger deviations of the surface potential the samples show significant response and/or drift. More than a correlation of structure, morphology, surface charge and electrochemical electrode properties is not possible at this stage, considering the highly complex nanoscale system. Nevertheless, our concept of spatial surface charge engineering is directly confirmed by this correlation, which successfully allowed to realize an InGaN-based all-solid-state reference electrode for electrochemical sensors.

## Conclusions

In conclusion, we have demonstrated a stable, epitaxial InN/InGaN-on-Si reference electrode for electrochemical sensors. A subtle interrelation of structure design, surface morphology and surface charge realized less than 10 mV/decade potential response in KCl aqueous solutions over a wide concentration range of 0.001 to 1 M and less than 2 mV/hour long-time drift in KCl aqueous solution with fixed concentration over 12 hours. This was made possible by our concept of spatial surface charge engineering, creating balanced nanoscale regions with positive and negative surface charge, such that a concentration insensitive and stable response originated. Paired with the InN/InGaN QD sensing electrode with solely changed structure and surface morphology, exhibiting super-Nernstian response, this opens the door to all-InGaN, all-solid-state, compact and robust electrochemical sensors with unprecedented performance.

## Methods

### Sample growth and characterization

Growth of the InN/InGaN structures was by plasma-assisted molecular beam epitaxy (PA-MBE) on p-type Si (111) substrates^47,48^. Active N was supplied by a radio-frequency (RF) plasma source. Prior to growth, the native oxide was removed from the Si surface by etching for 1 minute in 10% HF, the substrates were degassed for 30 min at 200 °C in the MBE load chamber and exposed to active N flux with 350 W RF power and 1.7 standard cubic centimeter per minute (sccm) N_2_ flow rate for 10 minutes at 800 °C in the MBE growth chamber. This surface nitridation results in improved crystal quality. Various growth temperatures and active N fluxes were adopted for InGaN and InN growth. For the two sets of samples explicitly discussed, the growth temperature was 510–520 °C for InGaN, close to the InGaN decomposition temperature, and the growth temperature for InN was 450 °C. The InGaN growth rate was 190 nm/hour, determined for compact, planar layers grown on GaN/sapphire substrates. The active N plasma source settings were 220–230 W and 1.2 sccm for an active N flux close to stoichiometric conditions. The InN deposition amount was between 0.25 and 1.2 monolayer (ML) and the InGaN layer thickness was between 35 and 190 nm, also referring to compact, planar layers on GaN/sapphire. The In content of the InGaN layer was 45%, determined by X-ray diffraction (XRD), shown in Supplementary Fig. S3. The surface morphology and cross-section were examined by top-view and cross-sectional scanning-electron microscopy (SEM). The relative surface potential was measured by Kelvin probe force microscopy (KPFM) using a Pt-Ir coated silicon tip. The spatial resolution was about 100 nm. Illumination was by a 3 W Hg discharge lamp. The original KPFM data are shown in Supplementary Fig. S4.

### Electrode fabrication

For electrode fabrication, the samples were diced into about 1 × 1 cm^2^ pieces. Ga-In eutectic was deposited on the Si substrate back side for ohmic contact formation. This takes advantage of the ohmic contact formed between p-Si and In-rich n-InGaN^49^ and the high n-type conductivity of the InGaN layer due to defects acting as donors. Silver paste was distributed over the Ga-In eutectic and Si substrate back side for mounting the samples on Cu stripes. The samples and Cu stripes were wrapped with electrically insulating Teflon tape with a 0.031 cm^2^ hole on the sample front side. A scheme of the growth and electrode fabrication is shown in Fig. S5.

### Open circuit potential measurements

The open circuit potential (OCP) was measured with the InN/InGaN electrodes and an Ag/AgCl reference electrode immersed in KCl aqueous solutions and connected to an electrochemical work station, see schematic drawing in Supplementary Fig. S6. As the Ag/AgCl electrode is a very good reference electrode, all measured potential changes are due to the InN/InGaN electrodes. For conditioning, the InN/InGaN electrodes were stored for three days in deionized water before the measurements. For sample selection, the InN/InGaN electrodes were alternately immersed for 150 seconds in 0.1 and 1 M KCl aqueous solutions. The most promising electrode was subjected to a wide KCl concentration range between 0.001 and 1 M and additionally tested for 12 hours in 0.1 M KCl aqueous solution to assess the long-time stability.

## Supplementary information

Supplementary Material

## Data Availability

The authors declare that the main data supporting the findings of this study are available within the paper and its Supplementary Information file. Other relevant data are available from the corresponding author upon reasonable request.
