# Supplementary material for: Spatial Surface Charge Engineering for Electrochemical Electrodes
Source: Sci Rep. 2019 Oct 10;9:14489. doi: 10.1038/s41598-019-51048-5 (PMC6787049; doi:10.1038/s41598-019-51048-5)
Supplement: Supplementary file 1 — Supplementary Material [file 41598_2019_51048_MOESM1_ESM.pdf]

Supplementary information

Spatial surface charge engineering for electrochemical electrodes

Lingyun Xie[1], Peng Wang[1], Yinping Qian[1], Lujia Rao[1], Hongjie Yin[1], Xingyu Wang[1], Hedong Chen[1], Guofu Zhou[1,2,3] & Richard Nötzel[1,2]

[1]*Guangdong Provincial Key Laboratory of Optical Information Materials and Technology, South China Academy of Advanced Optoelectronics, South China Normal University, Guangzhou 510006, P. R. China.*
[2]*National Center for International Research on Green Optoelectronics, South China Normal University, Guangzhou 510006, P. R. China.*
[3]*Academy of Shenzhen Guohua Optoelectronics, Shenzhen 518110, P. R. China*

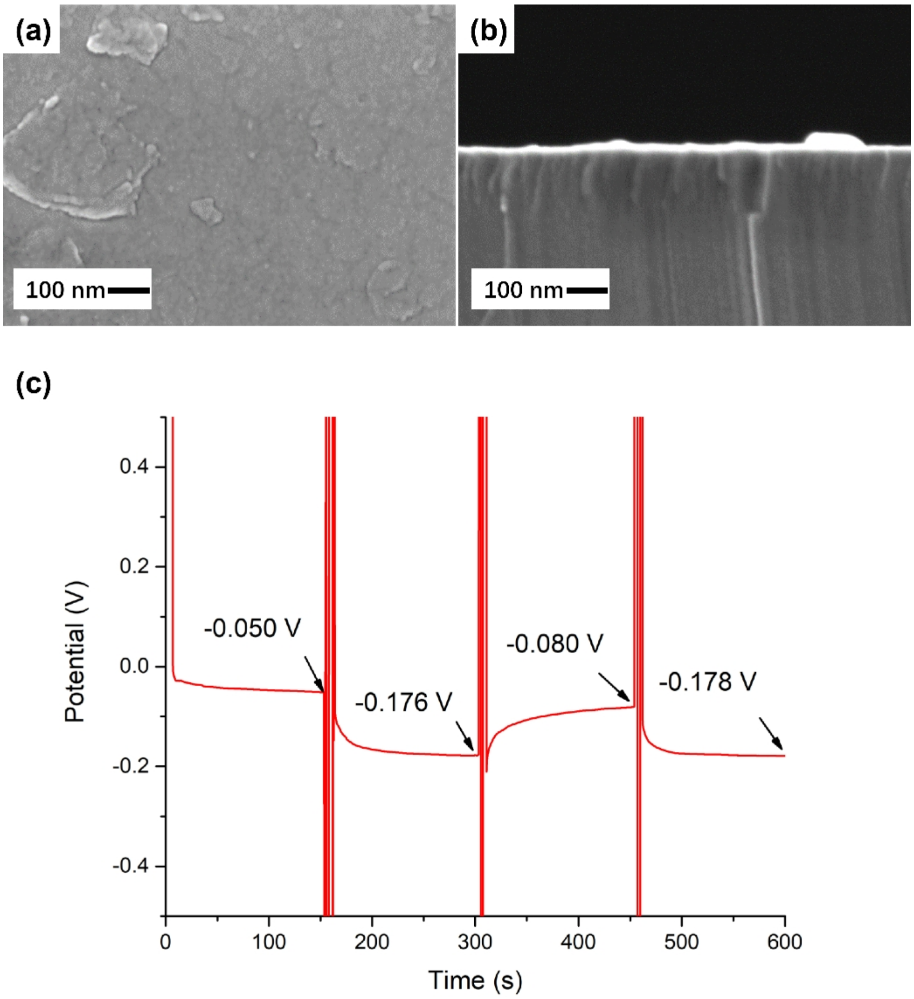

Figure S1. InN/InGaN structure on GaN/sapphire at the critical thickness of quantum dot formation and open circuit potential. (a) Top-view and (b) cross-sectional SEM images of the 0.8-ML-InN/130-nm-$In_{0.45}Ga_{0.55}N$ sample grown on a GaN/sapphire substrate. Onset of QD formation is observed. (c) OCP as a function of time for the InN/InGaN electrode and Ag/AgCl reference electrode immersed alternately for 150 seconds in 0.1 and 1 M KCl aqueous solutions.

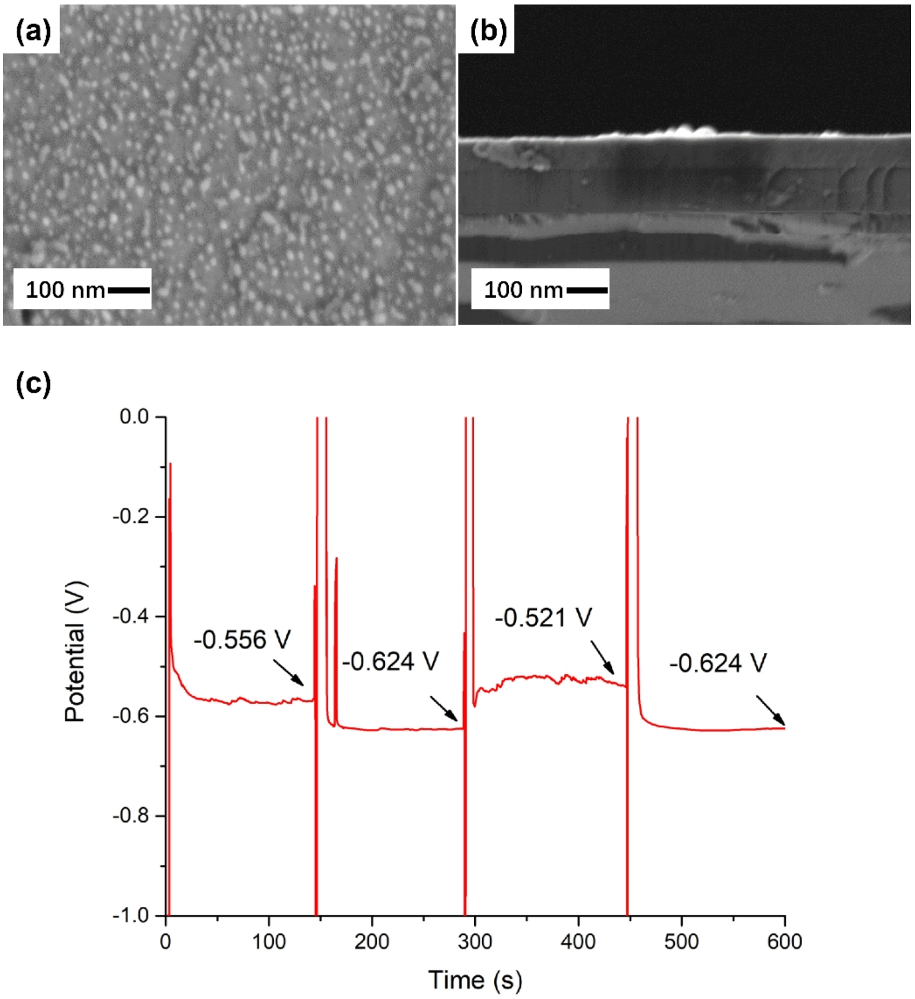

Figure S2. InN/InGaN structure on GaN/sapphire with clear quantum dot formation and open circuit potential. (a) Top-view and (b) cross-sectional SEM images of the 1.2-ML-InN/130-nm-$In_{0.45}Ga_{0.55}N$ sample grown on a GaN/sapphire substrate. Clear QD formation is observed. (c) OCP as a function of time for the InN/InGaN electrode and Ag/AgCl reference electrode immersed alternately for 150 seconds in 0.1 and 1 M KCl aqueous solutions.

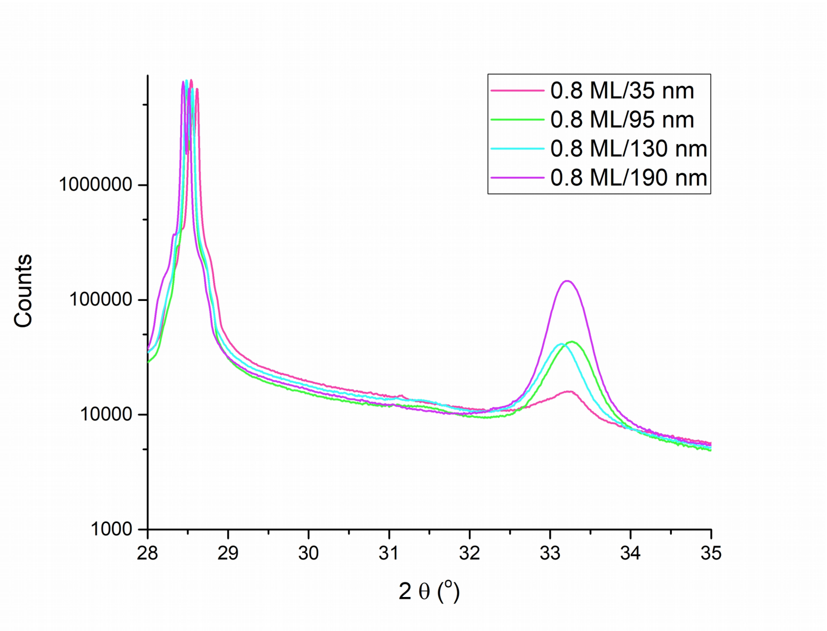

Figure S3. XRD spectra recorded around the symmetric Si (111) Bragg reflection of samples: 0.8-ML-InN/35-nm-InGaN (red line), 0.8-ML-InN/95-nm-InGaN (green line), 0.8-ML-InN/130-nm-InGaN (blue line), and 0.8-ML-InN/190-nm-InGaN (violet line).

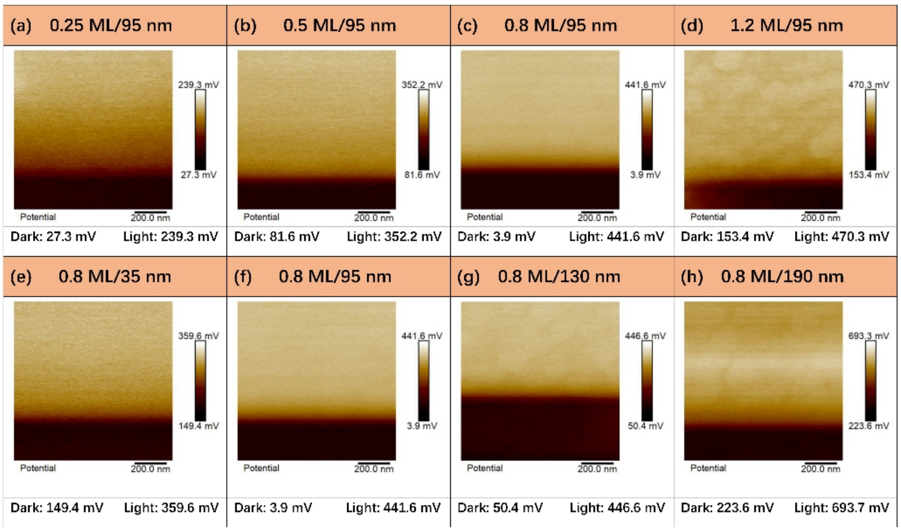

Figure S4. KPFM images and relative surface potentials of samples (a) 0.25-ML-InN/95-nm-InGaN, (b) 0.5-ML-InN/95-nm-InGaN, (c) 0.8-ML-InN/95-nm-InGaN, (d) 1.2-ML-InN/95-nm-InGaN, (e) 0.8-ML-InN/35-nm-InGaN, (f) 0.8-ML-InN/95-nm-InGaN, (g) 0.8-ML-InN/130-nm-InGaN, and (h) 0.8-ML-InN/190-nm-InGaN.

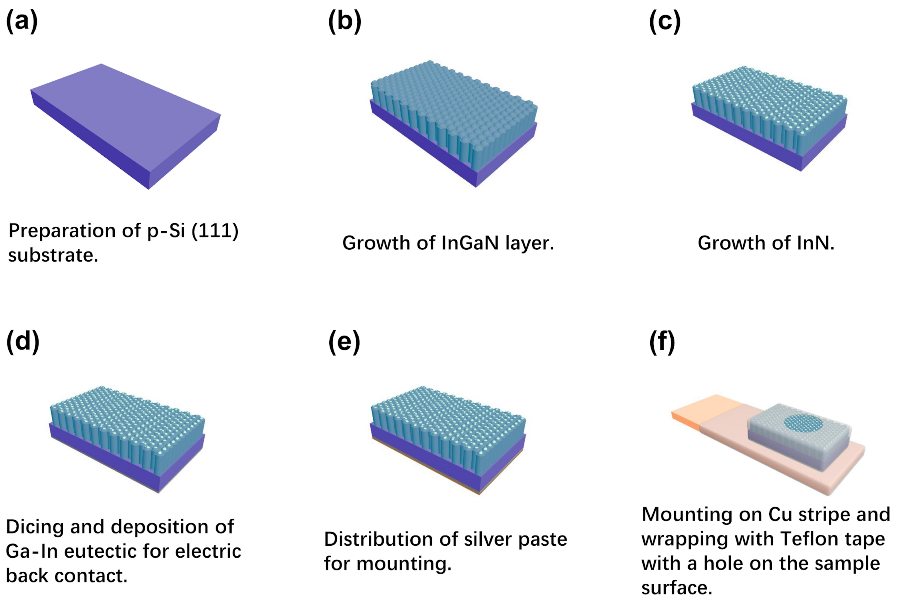

Figure S5. Schematic drawings of the (a-c) growth process and (d-f) electrode fabrication.

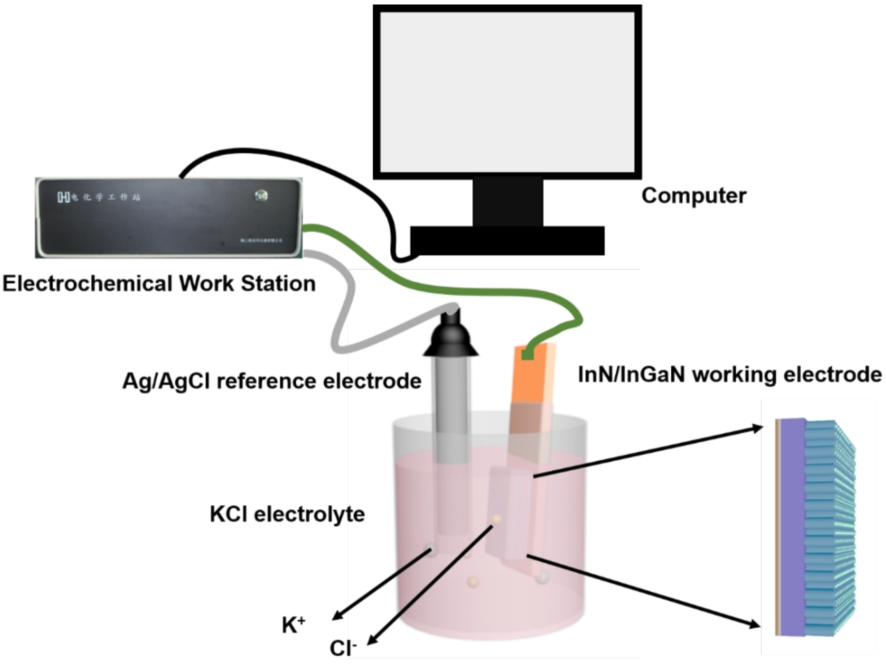

Figure S6. Schematic drawing of the measurement setup.
